# The clinical spectrum of ataxia telangiectasia in a cohort in Sweden

**DOI:** 10.1016/j.heliyon.2024.e26073

**Published:** 2024-02-15

**Authors:** Hannes Lindahl, Eva Svensson, Annika Danielsson, Andreas Puschmann, Per Svenningson, Bianca Tesi, Martin Paucar

**Affiliations:** a Department of Clinical Immunology and Transfusion Medicine, Karolinska University Hospital, Stockholm, Sweden; b Department of Clinical Neuroscience, Karolinska Institutet, Stockholm, Sweden; c Department of Pediatric Neurology, Karolinska University Hospital, Stockholm, Sweden; d Department of Pediatric Neurology, Sachska Children's Hospital, Stockholm, Sweden; e Neurology, Department of Clinical Sciences Lund, Lund University, Sweden; f Skane University Hospital, Lund, Sweden; g SciLifeLab National Research Infrastructure, Sweden; h Department of Neurology, Karolinska University Hospital, Stockholm, Sweden; i Department of Clinical Genetics and Genomics, Karolinska University Hospital, Stockholm, Sweden; j Department of Molecular Medicine and Surgery, Karolinska Institutet, Stockholm, Sweden; k Department of Medicine, Center for Hematology and Regenerative Medicine, Karolinska Institutet, Stockholm, Sweden

**Keywords:** Ataxia telangiectasia, Case series, Movement disorders, Cancer, Immunodeficiency

## Abstract

Ataxia telangiectasia (A-T), caused by biallelic variants in the *ATM* gene, is a multisystemic and severe syndrome characterized by progressive ataxia, telangiectasia, hyperkinesia, immunodeficiency, increased risk of malignancy, and typically death before the age of 30. In this retrospective study we describe the phenotype of 14 pediatric and adult A-T patients evaluated at the Karolinska University Hospital in Sweden during the last 12 years. Most of the patients in this cohort were severely affected by ataxia and wheelchair use started at a median age of 9 years. One patient died before the age of 30 years, but five patients had survived beyond this age. Four patients received prophylactic immunoglobulin replacement therapy due to hypogammaglobulinemia and respiratory complications ranged from mild to moderate severity. Three patients developed type 2 diabetes in young adulthood and nine patients (64%) had a history of elevated liver function tests. Four patients were diagnosed with cancer at ages 7, 41, 47, and 49 years. All the *ATM* variants in these patients were previously reported as pathogenic except one, c.6040G > A, which results in a p.Glu2014Lys missense variant. With increased life expectancy, A-T complications such as diabetes type 2 and liver disease may become more common. Despite having severe neurological presentations, the A-T patients in this case series had relatively mild infectious and respiratory complications.

## 1 Introduction

Ataxia telangiectasia (A-T; MIM 208900) is a rare autosomal recessive disease caused by variants in the ataxia-telangiectasia mutated (*ATM*) gene [1]. *ATM* codes for a protein kinase that has a central role in DNA repair and cell cycle control. Absent ATM kinase activity leads to genomic instability, which is clinically manifested as early onset progressive ataxia, oculocutaneous telangiectasia, increased risk of malignancy, and typically death before the age of 30 [2]. Other manifestations include hyperkinesias (dystonia and chorea), axonal neuropathy, endocrine abnormalities, and variable respiratory symptoms. V(D)J-recombination during lymphocyte development relies on double strand break repair of immunoglobulin and T cell receptor genes. Loss of ATM kinase activity therefore also results in impaired B and T lymphocyte function and thus variable immunodeficiency [3]. Additionally, partial loss of ATM activity correlates with a milder clinical presentation, referred to as variant A-T (vA-T). Cancer and respiratory tract infection (RTI) are the leading causes of death in, respectively, classical A-T and vA-T [4,5]. Importantly, female heterozygous carriers of pathogenic *ATM* variants have an increased risk of breast cancer and may be offered additional breast cancer surveillance [6].

There is currently no accepted disease modifying treatment available but reaching a diagnosis is crucial for proper clinical management. Current guidelines for patients with A-T recommend avoiding ionizing radiation, vigilant monitoring for malignancy and endocrine diseases, preventing and treating RTIs, monitoring of lung function, and multidisciplinary rehabilitation therapy [7]. Increased awareness of the clinical variability of A-T, in particular regarding presenting symptoms, may reduce both diagnostic delay and morbidity [8]. This retrospective study aims to describe the current phenotypic and genotypic variability of A-T patients seen at Karolinska University Hospital and to explore genotype-phenotype correlations.

### 1.1 Patients and methods

Clinical data were retrospectively collected from medical records for all pediatric and adult individuals affected by A-T or vA-T that had been evaluated at the Karolinska University Hospital during the last 12 years excluding those for whom we lack study consent (n = 1). Notably, A-T patient care is not centralized to one institution in Sweden and this is not a national cohort. A-T was diagnosed according to established guidelines [9]. Clinical assessment was performed by neurologists specialized in movement disorders. Disease severity was assessed using the Scale for Assessment and Rating of Ataxia (SARA), which ranges from score 0 (no ataxia) to 40 [10]. In selected cases ATM protein level was assessed using Western blot on lysates of a lymphoblastoid cell line prepared from the patient's blood [11]. Patient p12 as well as the unusual presentation with generalized dystonia in patient p13 have been previously reported [12,13]. Data on carriership status of relatives with cancer is unfortunately not available. The HGVS nomenclature of the reported *ATM* variants is based on the reference transcript NM_000051.4. *ATM* variants were classified according to American College of Medical Genetics and Genomics guidelines [14].

## 2 Results

### 2.1 Demographic features and diagnosis

**Fig. 1:**
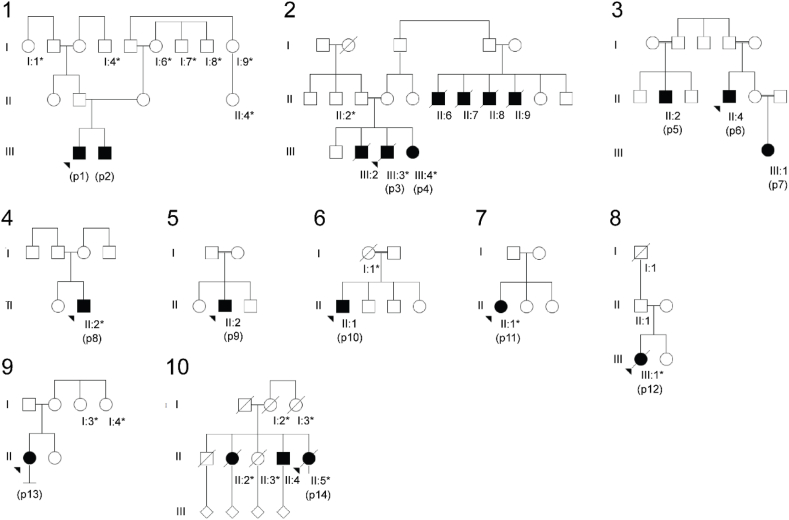
Pedigrees of the included families. Families 1–8 include the 12 patients with classical A-T and families 9–10 the two patients with vA-T. Squares indicate males and circles females. Ataxia is indicated by a filled shape (includes both diagnosed A-T as well as relatives living outside of Sweden with ataxia-like movement disorder based on descriptions provided by family members), cancer by an asterisk, and the index case by an arrowhead. Diagonal line indicates deceased individual.

Fourteen affected individuals (8 males and 6 females) from 10 different families are included in this study (Fig. 1 and Table 1). Twelve patients have been classified as classical A-T and two as vA-T. Three died at age 28, 50, and 51, respectively. Age at last visit ranged from four to 51 years (median 28, IQR 17–46) at the time of patient records review. The families are described in more detail in the article's supplementary materials.

**Table 1:** Demographic data.

Patient	sex	ethnicity	age of onset	age of diagnosis	diagnosis	current age/age of death*	
p1^family1^	M	Swedish	1	2	A-T	6	
p2^family1^	M	Swedish	3	0.3	A-T	4	
p3^family2^	M	Syrian	3	25	A-T	28*	
p4^family2^	F	Syrian	3	18	A-T	23	
p5^family3^	M	Kurdish	<1	4	A-T	17	
p6^family3^	M	Kurdish	1	7	A-T	29	
P7^family3^	F	Kurdish	2	4	A-T	17	
P8^family4^	M	Swedish	<1	<1	A-T	8	
p9^family5^	M	Turkish	early childhood	NA	A-T	23	
p10^family6^	M	Turkish	3	NA	A-T	35	
p11^family7^	F	Swedish	preschool age	11	A-T	46	
p12^family8^	F	Swedish	1	22	A-T	51*	
p13^family9^	F	Lithuanian	childhood	36	vA-T	46	
p14^family10^	F	Chilean	7	45	vA-T	50*	

A-T, ataxia telangiectasia; vA-T, variant A-T. Age and time is indicated in years.

### 2.2 Neurologic features

Median age of onset was 2.5 years in the 12 patients with classical A-T but the patients with vA-T had later onset (Table 1). Notably, the median time from onset to diagnosis was 5 years. In all cases except three the presenting features included unsteady gait, balance disturbance, or ataxia (Table 2). Patient p2 presented with isolated speech difficulty which, at age four, still is the dominating feature. His older brother (p1) is the only patient in this case series that has needed tube feeding, which started at age 2. Patient p13 presented with involuntary movements. Patient p8 was identified in the national newborn screening program for severe combined immunodeficiency due to low lymphocyte counts at birth. He then developed balance disturbance before age one. Ataxia was present in all patients except one (p13) and with increased severity in those with classical A-T as shown by a median SARA score of 30 for these 12 patients compared to a median score of nine for the two with vA-T. Dystonia was present in 11/14 (79%) and chorea was present in 6/14 (43%). Polyneuropathy, determined by clinical examination was present in 9/13 (69%) and by neurophysiological studies in 3/5 (60%), cognitive impairment in 8/14 (53%), wheelchair use in 11/14 (79%), and cerebellar atrophy in 9/11 (82%) patients. Polyneuropathy and cognitive impairment were only observed in patients with classical A-T. Two patients displayed a phenotype compatible with vA-T of which one (p13) developed generalized dystonia [12] and the other (p14) relatively mild ataxia and has managed university level studies.

**Table 2:** Neurologic features.

patient	presenting features	ataxia	other movement disorder	clinical polyneuropathy	neurophysiology	cognitive impairment	wheelchair use from age, y	SARA	MRI findings	age at MRI
p1^family1^	unsteady gait	yes	dystonia, chorea	no	NA	no	no	NA	normal	2
p2^family1^	speech difficulty	yes	no	no	NA	no	no	NA	NA	NA
p3^family2^	balance disturbance	yes	dystonia	yes	NA	yes	4	34	severe atrophy of vermis and cerebellar hemispheres	27
p4^family2^	balance disturbance	yes	dystonia, myoclonus	yes	Abnormal ENeG	severe	4	NA	severe atrophy of vermis and cerebellar hemispheres, cavernoma	17
p5^family3^	balance disturbance	yes	dystonia	yes	Abnormal ENeG	yes	8	20	moderate atrophy of vermis and cerebellar hemispheres	10
p6^family3^	delayed speech and motor development	yes	no	yes	NA	yes	10	28	NA	NA
P7^family3^	balance disturbance	yes	dystonia, dyskinesia, myoclonus	yes	NA	yes	17	24	general cerebellar atrophy, arachnoid cyst	8
P8^family4^	immunodeficiency	yes	dystonia, chorea	not described	NA	no	6	NA	severe general cerebellar atrophy	8
p9^family5^	ataxia	yes	dystonia	yes	NA	yes	10	29	severe general cerebellar atrophy	11
p10^family6^	ataxia	yes	dystonia, chorea, myoclonus	yes	Abnormal ENeG	yes	9	31	severe general cerebellar atrophy	26
p11^family7^	unsteady gait	yes	dystonia, chorea	yes	NA	no	yes	33	NA	NA
p12^family8^	unsteady wide gait	yes	chorea	yes	NA	yes	10	37.5	vermis atrophy^a^	10
p13^family9^	involuntary movements	no	dystonia, myoclonus	no	Normal ENeG and EMG	no	yes	10	normal	35
p14^family10^	balance disturbance, dysarthria	yes	dystonia, chorea, myoclonus	no	Normal ENeG and EMG	no	no	7.5	mild vermis atrophy	44

a SARA, Scale for the assessment and rating of ataxia; MRI, magnetic resonance imaging; ENeG, electroneurography; EMG, electromyography.

### 2.3 Non-neurologic features

Telangiectasias were present in 12/14 (86%) of the patients (Table 3). Short stature, here defined as more than 2 standard deviations below age-adjusted average height at latest assessment, was present in 6/13 (46%) patients (Table 3). However, the adolescent growth spurt has yet to occur in p1, p2, and p8, all currently with normal height. At least one endocrine disease was present in 5/14 (36%) and included three cases of type 2 diabetes, two cases of hypothyroidism, one case of hyperthyroidism, and one case of prolactinoma.

**Table 3:** Non-neurologic features.

patient	telangiectasias	short stature	BMI (Z-score)	endocrine disease	respiratory symtoms	malignancy	age at first cancer, y	other diagnoses
p1^family1^	yes	no	16 (+0.5)	no	non-allergic asthma	no		vitiligo, laryngomalacia
p2^family1^	yes	no	17 (0)	no	persistent cough	no		laryngomalacia
p3^family2^	yes	yes	15	no	no	no		scoliosis, constipation
p4^family2^	no	yes	21	hypothyroidism, diabetes type 2	no	no		hepatic steatosis, primary biliary cholangitis
p5^family3^	yes	yes	27 (+1.5)	no	no^a^	no		no
p6^family3^	yes	yes	29	no	no	no		constipation
P7^family3^	no	yes	18 (−2)	no	no	no		constipation
P8^family4^	yes	no	13 (−2.5)	no	mild respiratory failure	DLBCL	7	immune thrombocytopenic purpura
p9^family5^	yes	yes	23	no	no	no		dysautonomia, Tietze syndrome
p10^family6^	yes	no	26	diabetes type 2	asthma	no		vitiligo, essential hypertension
p11^family7^	yes	no	25	hyperthyroidism	restrictive lung disease^a^	thyroid, vulva, anal cancer	41	no
p12^family8^	yes	no	23	no	no	colon cancer	49	no
p13^family9^	yes	NA	NA	no	no	no		no
p14^family10^	yes	no	21	hypothyroidsim, prolactinoma	asthma, suspected restrictive lung disease^a^	pancreas cancer	47	no

a Body mass index (BMI) is the most recent recorded for each patient. Z-scores are proved for pediatric patients. DLBCL, Diffuse large B cell lymphoma.

A history of respiratory symptoms or abnormal lung function test results were present in 6/14 (43%) of the patients. One had a restrictive lung disease (p11) and one had mild respiratory failure and was intermittently treated with oxygen (p8). In the other four patients the respiratory manifestations consisted of relatively mild obstructive symptoms.

One patient (p11) was diagnosed with three different forms of cancer (thyroid, vulva, and anal) between age 41 and 43 years. Three additional patients were diagnosed with cancer before the age of 50. Taken together, 3/4 of patients aged 40 or more had at least one form of cancer. Patient p12 had had a prophylactic mastectomy, had gone through a hysterectomy due to a myoma, and died from colon cancer. Notably, cancer was not restricted to patients with classical A-T. Only one patient in our cohort had hematologic malignancy, lymphoma, diagnosed at age 7 years. In no case was cancer treated with radiation in this cohort due to the hypersensitivity to ionizing radiation inherent to A-T. Chemotherapy was used, but in the case of p8 with reduced dose. In three of these families there were clusters of various cancer types (Family 1, 3 and 10) among non-ataxia relatives (Fig. 1 and Supplementary Materials).

Autoimmune disease was present in 4/14 (29%) of the patients and included two cases of vitiligo, one case of primary biliary cholangitis, and one case of immune thrombocytopenic purpura. Additionally, Tietze syndrome, an inflammatory disorder of unclear etiology, also occurred in one of the patients and autoimmunity in the form of anti-glutamic acid decarboxylase (GAD)-antibodies with no apparent disease manifestation in another one.

#### 2.3.1 Immunologic and laboratory features

All patients but the one (p6) had elevated alpha-fetoprotein at first sampling (range 14–1200 μg/L) with a median value of 116 μg/L (Table 4). Patient p6 had normal alpha-fetoprotein at first sampling (17 μg/L at age five) but at the age of seven years it was slightly above normal and at age 16 it was clearly elevated (40 μg/L).

**Table 4:** Laboratory and immunologic features.

patient	IgG	IgM	IgA	IgG_1_	IgG_2_	IgG_3_	IgG_4_	neutrophils	T cells	B cells	CD4 T cells	CD8 T cells	T cell proliferation	AFP	elevated liver enzymes	other laboratory abnormalities	infection history	IgRT
p1^family1^	4.13†	0.21†	<0.08†	2.7†	0.05†	0.09†	<0.01	3100	550†	60†	280†	200†	normal	95*	yes		–	yes
p2^family1^	0.76†	0.15†	<0.08†					1500†	190†	60†	140†	30†	normal	175*	yes		I	yes
p3^family2^	16.8*	2.27*	<0.07†	13.6	1.35	0.91	0.17	6800*	640†	40†				909*	no	eosinophilia, poor vaccine response	–	no
p4^family2^	10	2.2*	<0.07†	7.91	1	0.42	0.28	5400	640†	40†	400†	210		1200*	yes	eosinophilia, poor vaccine response, microcytic anemia	–	no
p5^family3^	13.6	1.89*	3.65*	7.65	2.3	1.38	0.77	2800	1030	80	710	300	normal	40*	yes		–	no
p6^family3^	9.29	1.37	1.33	6.67	1.89	1.12	0.26							17	no		II	no
P7^family3^	9.52	1.23	1.57	5.54	3.04	0.76	0.38	3300	1130	130	630	370	normal	14*	no		–	no
P8^family4^	7.8	0.14†	<0.08†	NA	NA	NA	NA	4300	290†	50†	230†	50†	normal	136*	yes		III	yes
p9^family5^	12.5	0.95	0.94	7.63	2.18	0.68	0.01	4500	1740	170	1070	580		58*	yes		–	no
p10^family6^	9.71	0.57	<0.07†	7.02	2.55	0.53	0.013	5400	2050	180	870	1000	normal	155*	yes	microcytic anemia, hypertriglyceridemia	IV	yes
p11^family7^	12.9	1.3	4.2	9.1	1.3	0.9	0.05	6300*						213*	yes	elevated T3, T4	V	no
p12^family8^	6†	5.5*	4.5											30*	no		VI	no
p13^family9^	15.4	2.9*	1.7	8.36	5.75	0.66	0.02	3300						75*	no		–	no
p14^family10^	18*	4.5*	2.5					3300						177*	yes	anti-GAD-antibodies	–	no

Immunoglobulin (Ig) levels are in g/L. Leukocyte levels are in cells/mm^3^. Alpha-fetoprotein (AFP) is in μg/L. Liver enzymes include transaminases, alkaline phosphatase, gamma-glutamyltransferase, and lactate dehydrogenase. Values that are above and below the laboratory age adjusted reference range are marked with * and †, respectively. Patients with a history that may suggest increased infectious susceptibility are denoted with roman numerals: (I) had recurrent episodes of low-grade fever (II) bacterial pneumonia, acute media otitis twice in adulthood (III) recurrent bacterial pneumonia (IV) 3–4 respiratory tract infections per year (V) bacterial pneumonia twice (VI) recurrent labial herpes. IgRT, Ig replacement therapy.

Six patients had a history that suggested increased infectious susceptibility, but only two were severely affected (p8 and p11), with bacterial pneumonia at least twice. Four patients had hypogammaglobulinemia of which three also had subnormal IgM. Four patients were on continuous immunoglobulin replacement therapy of which three were treated because of hypogammaglobulinemia and one (p10) due to IgA-deficiency with frequent RTIs. This patient with IgA-deficiency has recently stopped IgRT because of good infectious disease control. In total, IgA-deficiency was present in six patients, including the two sibling pairs. Immunoglobulin deficiencies were only observed in patients with classical A-T. No subclass specific IgG deficiencies were present.

The two sibling pairs all had combined B and T cell deficiency. CD4 T cells appeared more affected than CD8 T cells and according to local guidelines trimethoprim/sulfamethoxazole prophylaxis was given to p2 and p8, who had markedly reduced CD4 T cells. One of these patients (p2) had a slight neutropenia in addition to a deficiency in B cells, CD4 T cells, CD8 T cells, IgG, IgM, and IgA and furthermore experienced recurring episodes of low-grade fever. Two patients (sibling pair p3 and p4) had unexplained eosinophilia. The same two patients had poor serological response to vaccinations despite normal and slightly elevated overall IgG levels.

Conspicuously, 9/14 (64%) of the patients had at least one occurrence of elevated liver enzymes (transaminases, alkaline phosphatase, gamma-glutamyltransferase, or lactate dehydrogenase). In one case, primary biliary cholangitis was diagnosed and in another one the values normalized after cessation of alcohol consumption. Moreover, two unrelated patients had microcytic anemia.

#### 2.3.2 ATM variants

Causative *ATM* variants were detected for all patients (Table 5). The patients with classic A-T either carried a homozygous (n = 7) or two heterozygous (n = 5) loss-of-function variants. Apart from p1 and p2, compound heterozygosity was not confirmed with parental samples or tests of siblings. The two patients with vA-T (p13 and p14) carried heterozygous loss-of-function variants and/or missense variants. In both cases, functional data demonstrated reduced ATM protein expression. One patient with vA-T (p14) had an *ATM* variant (c.6040G > A, p.Glu2014Lys) not previously reported in classical A-T or vA-T. However, the G > T variant in the same position has been linked to classical A-T [15]. Five patients from three families were homozygous for c.3576G > A [16], which makes it the most common variant in this cohort. All carriers of this variant were of Turkish or Kurdish ethnicity, but no clear disease phenotype correlation could be observed.

**Table 5:** ATM variants.

patient	consanguineous parents	affected siblings	cDNA change	protein change	variant type	ACMG classification	ATM protein (activity)	reference
p1-2^family1^	no	sibling with A-T	c.8655dup c.332-?_8850+?del	p.Val2886Cysfs*10 NA	truncation full gene deletion	5 5	NA	[29]
p3-4^family2^	yes	3 siblings: A-T, suspected A-T, and not affected	c.7788G > A homozygous	p.Glu2596Glu	aberrant splicing	5	very low (absent)	[16]
p5-7^family3^	no	2 (p5), 3 (p6), 1 (p7) siblings, none affected	c.3576G > A homozygous	p.Lys1192Lys	aberrant splicing	5	NA	[16]
P8^family4^	no	1 sibling, not affected	c.3673C > T c.8655dup	p.Gln1225* p.Val2886Cysfs*10	truncation truncation	5 5	NA	[29] [29]
p9^family5^	yes	2 siblings, not affected	c.3576G > A homozygous	p.Lys1192Lys	aberrant splicing	5	NA	[16]
p10^family6^	yes	3 siblings, not affected	c.3576G > A homozygous	p.Lys1192Lys	aberrant splicing	5	NA	[16]
p11^family7^	no	2 siblings, not affected	c.9029T > G c.6095G > A	p.Leu3010Ter p.Arg2032Lys	truncation aberrant splicing	4 5	NA	[30] [15]
p12^family8^	no	1 sibling, not affected	c.487C > T c.3284G > C	p.Gln163* p.Arg1095Thr	truncation aberrant splicing	5 4	NA	[30] [30]
p13^family9^	no	1 sibling, not affected	c.3214G > T c.8147T > C	p.Glu1072* p.Val2716Ala	truncation missense	5 5	reduced (normal)	[12] [12]
p14^family10^	no	4 siblings, 2 with undiagnosed movement disorder	c.6040G > A c.8122G > A	p.Glu2014Lys p.Asp2708Asn	missense missense	3 5	reduced (reduced)	NA [31]

*ATM* (NM_000051.4) variants including American College of Medical Genetics (ACMG) criteria classification. ATM protein expression was assessed *in vitro*. A-T, ataxia telangiectasia; ATM, A-T mutated.

## 3 Discussion

Herein we describe the current spectrum of A-T patients at a tertiary care center in Sweden. Adherence to A-T management guidelines contributes to the prevention of complications and reduces the burden of disease [7]. The type and severity of manifestations observed in individuals affected by A-T can be expected to change with improved health care [17]. Of note is that survival beyond age 30 years is possible despite the presence of severe neurological features. With advanced age, complications such as diabetes mellitus type 2 and liver disease are expected to become more common and warrant more attention [17,18].

The most common presenting feature in classical A-T is cerebellar ataxia [19], which was also the case in our cohort. Moreover, dystonia is also a feature for both classical A-T and vA-T [20], and present in some of our patients. One of the patients with vA-T (p13), who was reported previously [12], presented with severe generalized dystonia but no ataxia. Interestingly, the brain MRI of this patient was normal. In classical A-T cerebellar atrophy develops in virtually all patients but in vA-T this may be less apparent. In fact, in a previously reported series of vA-T cases that, similar to p13, presented with dystonia without prominent ataxia there was no cerebellar atrophy on MRI [22].

Consistent with previous reports, vA-T was not associated with overt immunological impairment [19]. Moreover, most patients with an immunodeficiency phenotype in this case series were not severely affected by infectious disease susceptibility or bronchiectasis. Three patients had a history of pneumonia but one of these (p6) had severe dysphagia, suggesting aspiration may have been the cause rather than immunodeficiency. Several case series have reported higher incidence of recurrent RTIs [23,24] but others have observed a relatively low infectious disease susceptibility in relation to laboratory immunologic abnormalities [25,26]. Although the prevalence of liver disease in A-T increases with age we here have observed elevated liver enzymes also in the youngest patients (p1, p2, and p8).

A-T patients with low IgG and IgA but with normal or elevated IgM have been referred to as hyper-IgM phenotype with hypogammaglobulinemia (AT-HIGM phenotype). This A-T subset is associated with distinctly worse prognosis and typically die before age 15 from respiratory failure [2]. Similarly, IgG2-deficiency is also associated with poor prognosis [2]. No patient in this cohort had AT-HIGM phenotype or isolated IgG2-deficency. The AT-HIGM phenotype does not seem to be associated with *ATM* genotype, which suggests that environment and possibly unknown modifiable factors may underlie this phenotype.^24^

A limitation with this study is the small size, which makes it difficult to draw conclusions regarding genotype-phenotype correlations. However, even with larger case series this has been challenging due to the large number of pathogenic variants [27,28]. Furthermore, ATM variants with reduced kinase activity underlie vA-T but we have assessed ATM protein levels and kinase activity in relation to only three genotypes and can therefore not formally exclude that patients other than p13 and p14 are also vA-T.

A-T is a complex incurable multisystem disease associated with poor prognosis. Knowledge and awareness of its wide variable phenotype and adherence to guidelines may improve this dire outcome.

## Ethical statement

This study was reviewed and approved by Swedish Ethical Review Authority with the approval number: Dnr 2016/2503-31/2. All patients or their legal guardians provided informed consent to participate in the study and for the publication of their anonymised case details.

## Study funding

Martin Paucar obtained funding from Region Stockholm, The 10.13039/100009389Promobilia Foundation, and NeuroSweden. Bianca Tesi obtained funding from Region Stockholm.

## Data availability statement

All original data for this study is included in the article and supplementary material.

## CRediT authorship contribution statement

**Hannes Lindahl:** Writing – original draft, Visualization, Formal analysis, Data curation. **Eva Svensson:** Writing – review & editing, Validation, Investigation. **Annika Danielsson:** Writing – review & editing, Validation, Investigation, Conceptualization. **Andreas Puschmann:** Writing – review & editing, Validation, Investigation. **Per Svenningson:** Writing – review & editing, Supervision, Conceptualization. **Bianca Tesi:** Writing – review & editing, Validation, Data curation. **Martin Paucar:** Writing – review & editing, Visualization, Validation, Project administration, Investigation, Funding acquisition, Conceptualization.

## Declaration of competing interest

The authors declare the following financial interests/personal relationships which may be considered as potential competing interests: Martin Paucar reports financial support was provided by Promobilia foundation. Martin Paucar reports financial support was provided by NeuroSweden. If there are other authors, they declare that they have no known competing financial interests or personal relationships that could have appeared to influence the work reported in this paper.
