# Supplementary material for: The clinical spectrum of ataxia telangiectasia in a cohort in Sweden
Source: Heliyon. 2024 Feb 15;10(4):e26073. doi: 10.1016/j.heliyon.2024.e26073 (PMC10884802; doi:10.1016/j.heliyon.2024.e26073)
Supplement: Multimedia component 1 [file mmc1.docx]

The clinical spectrum of ataxia telangiectasia in a cohort in Sweden

Lindahl et al.

*Supplementary material*

Here follows a summary of the families reported:

**Family 1:** These two Swedish siblings, born to non-consanguineous parents, are members of a kindred with seven patients affected by different types of solid tumors. I:9 and her daughter (II:4) were diagnosed with breast cancer. I:6 had breast cancer in situ. I:1 had cervix cancer and I:4 had bladder cancer whereas I:7 and I:8 had prostate and skin cancer, respectively.

**Family 2:** This consanguineous kindred from Syria includes 7 individuals affected by ataxia. We did not have any medical records on patients II:6, II:7, II:8, or II:9 and III:2 who died between ages 18-20 years. Patient II:2 was diagnosed with colon cancer.

**Family 3:** This consanguineous Kurdish family from Turkey includes 3 patients included in this case series (Patients 5, 6 and 7) with ataxia. We did not have any records of malignancy among relatives.

**Family 4:** This is a Swedish family in which the index case (p8, II:2 in the pedigree) was also affected by B cell lymphoma. There are no other individuals with cancer in this family.

**Family 5:** In this consanguineous Turkish family, there were not recorded cases of malignancy.

**Family 6:** In this consanguineous Turkish family the index case’s mother (I:1) died of colon cancer.

**Family 7:** The index case (p11, II:1) in this non-consanguineous Swedish family suffered from three different types of cancer.

**Family 8:** The index case (p12, III:1) in this non-consanguineous Swedish family died from widespread colon cancer. Both her father and paternal grandfather (II:1 and I:1) had prostate cancer.

**Family 9:** This Lithuanian patient (p13) has two maternal aunts who suffered from malignancies (breast and gastric cancer).

**Family 10:** The index case (p14, II:5 in the pedigree), we do not have clinical notes on other members of this Chilean family. According to the index case, I:2 suffered from liver cancer and recurrent infections, I:3 died of lung cancer. II:2 was affected by ataxia and died of cervical cancer, whereas II:3 died from leukemia. The index case died from pancreatic cancer.
